# NKG2D Enhances Double-Negative T Cell Regulation of B Cells 

**DOI:** 10.3389/fimmu.2021.650788

**Published:** 2021-06-16

**Authors:** Shi-hua Hu, Long-hui Zhang, Jie Gao, Jing-heng Guo, Xiao-dong Xun, Xiao Xiang, Qian Cheng, Zhao Li, Ji-ye Zhu

**Affiliations:** ^1^ Department of Hepatobiliary Surgery, Peking University Organ Transplantation Institute, Peking University People’s Hospital, Beijing, China; ^2^ Peking University Centre of Liver Cancer Diagnosis and Treatment, Beijing Key Surgical Basic Research Laboratory of Liver Cirrhosis and Liver Cancer, Peking University People’s Hospital, Beijing, China

**Keywords:** CD4^-^CD8^-^ double-negative T cells, TCRαβ, MHC class II, NKG2D, dendritic cells, CD4^+^ T cells, B cells

## Abstract

Numerous studies reported a small subpopulation of TCRαβ^+^CD4^-^CD8^-^ (double-negative) T cells that exert regulatory functions in the peripheral lymphocyte population. However, the origin of these double-negative T (DNT) cells is controversial. Some researchers reported that DNT cells originated from the thymus, and others argued that these cells are derived from peripheral immune induction. We report a possible mechanism for the induction of nonregulatory CD4^+^ T cells to become regulatory double-negative T (iDNT) cells *in vitro*. We found that immature bone marrow dendritic cells (CD86^+^MHC-II^-^ DCs), rather than mature DCs (CD86^+^MHC-II^+^), induced high levels of iDNT cells. The addition of an anti-MHC-II antibody to the CD86^+^MHC-II^+^ DC group significantly increased induction. These iDNT cells promoted B cell apoptosis and inhibited B cell proliferation and plasma cell formation. A subgroup of iDNT cells expressed NKG2D. Compared to NKG2D^-^ iDNT cells, NKG2D^+^ iDNT cells released more granzyme B to enhance B cell regulation. This enhancement may function *via* NKG2D ligands expressed on B cells following lipopolysaccharide stimulation. These results demonstrate that MHC-II impedes induction, and iDNT cells may be MHC independent. NKG2D expression on iDNT cells enhanced the regulatory function of these cells. Our findings elucidate one possible mechanism of the induction of peripheral immune tolerance and provide a potential treatment for chronic allograft rejection in the future.

## Introduction

Approximately two decades ago, a novel subset of TCRαβ^+^CD3^+^CD4^-^CD8^-^ double-negative T (DNT) cells was identified in the peripheral lymphoid tissues of normal rodents and humans (1). These DNT cells lack the expression of CD4, CD8 and NK1.1 but express TCRαβ and CD3. Although these cells account for only 1% to 5% of the peripheral lymphocyte population, they play an important role in the induction of peripheral immune tolerance and participate in the regulation of inflammatory responses. For example, DNT cells significantly prolong allo- and xenogeneic graft survival, alleviate graft-versus-host disease, and prevent autoimmune diseases and cancer in an antigen-specific manner (2–8). Nonetheless, the origin of these DNT cells is controversial. Some researchers reported that DNT cells originated from the thymus (9), and others argued that these cells are derived from peripheral immune induction (10).

Previous researchers reported that nonregulatory CD4^+^ T cells might be induced to become DNT cells *in vitro* (11). These ex vivo-induced DNT cells (iDNT cells) exhibit a phenotype that is consistent with the physiological DNT cell phenotype and perform regulatory functions, including antigen-specific inhibition of T cell- and B cell-mediated immune responses (11–13). Nevertheless, the mechanisms of induction are not well understood.

Natural killer group 2-member D (NKG2D) is an activating receptor that is commonly expressed on all NK cells. NKG2D is also expressed on some subsets of NKT cells, activated murine CD8^+^ T cells, activated human CD8^+^ T cells, γδ T cells, murine macrophages, and a minor population of human CD4^+^ T cells (14–19). NKG2D is a fundamental receptor that binds to a variety of stress ligands, including ULBP and human Rae1 in humans. Rae1, H60 and Mult1 are the ligands of NKG2D in mice (20). The binding of NKG2D to its ligands induces NK cells to secrete cytokines that promote killing activity. NKG2D on CD8^+^ T cells acts as a costimulatory factor, and its binding leads to effector and memory T cell formation (21, 22). However, little is known about whether iDNT cells express NKG2D and bind to NKG2D ligands.

The present study examined the possible mechanisms of the induction of nonregulatory CD4^+^ T cells to become iDNT cells *in vitro*. We also examined the regulatory effects of iDNT cells on B cells and the mechanism of iDNT cell regulation of B cells. Our results suggested that immature bone marrow dendritic cells (CD86^+^MHC-II^-^ DCs), rather than mature bone marrow DCs (CD86^+^MHC-II^+^), induce iDNT cells. MHC-II impedes induction, and iDNT cells may be MHC independent. We also found that a group of iDNT cells expresses NKG2D. These NKG2D^+^ iDNT cells had a stronger ability to regulate B cells *via* NKG2D ligands than cells that did not express NKG2D.

## Materials and Methods

### Mice

Male 6-week-old C57BL/6 (H-2^b^) and BALB/c (H-2^d^) mice were purchased from Beijing Vital River Laboratory Animal Technology Co., Ltd. and maintained in specific pathogen-free animal facilities of Peking University People’s Hospital. The Peking University People’s Hospital Animal Ethics and Experimental Committee approved all animal experiments.

### Reagents and Flow Cytometry

Lipopolysaccharide (LPS) was obtained from Sigma (USA, California). Recombinant mouse granulocyte-macrophage colony-stimulating factor (GM-CSF), IL-2 and IL-4 were obtained from PeproTech (USA). A quantitative polymerase chain reaction (q-PCR) kit was purchased from Invitrogen. To analyze single-cell suspensions of lymphocytes, antibodies against CD3 (17A2), CD4 (GK1.5), CD8 (53-6.7), CD127 (A7R34), TCRβ (H57-597), and CD25 (7D4) were used to distinguish T cells. Antibodies against CD19, IgM and IgD were used to sort naïve B cells. An anti-CD40 antibody was used to stimulate naïve B cells (BioLegend, USA). Anti-CD86 (GL7), anti-MHC-II (I-A/I-E), and anti-CD11c (N418) antibodies were used to sort mature bone marrow DCs. Anti-CD3 (17A2), anti-CD4 (GK1.5), anti-TCRβ (H57-597) and anti-NKG2D (CX5) antibodies were used to characterize iDNT cells. Anti-Rae1 (186107), anti-ULBP-1/MULT-1 (FAB2588R-100UG) antibodies were used to detect the protein expression of Rae1 and Mult1 on B cells.

### Purification and Induction of Bone Marrow DCs

DCs isolated from mouse bone marrow were induced according to the methods of Lutz (23). Briefly, red blood cells were lysed, and bone marrow cells from male BALB/c mice were cultured with recombinant GM-CSF (10 ng/ml) and recombinant IL-4 (10 ng/ml) and treated with LPS (10 μg/ml) on day 6. Bone marrow DCs were harvested on day 7 *via* positive selection for CD11c, CD86 and/or MHC-II.

### Preparation of iDNT Cells *In Vitro*

iDNT cells were prepared as previously described with minor modifications (11). Briefly, CD4^+^CD127^hi^CD25^-^ T cells were obtained from the spleens of male C57BL/6 mice *via* flow cytometry sorting. The sorted T cells were cocultured with bone marrow DCs from BALB/c mice at a ratio of 1×10^5^ T cells to 2.5×10^4^ DCs for up to 7 days in 96-well round-bottom plates in complete RPMI 1640 (RPMI medium containing 10% FBS, 100 IU/ml penicillin, 100 μg/mL streptomycin and 2 mM L-glutamine). Different concentrations of recombinant IL-2 (0, 50 ng/mL, 100 ng/mL or 200 ng/mL) were added to the mixed lymphocyte reaction (MLR) to assess the induction effect of IL-2. TCRαβ^+^CD3^+^CD4^-^CD8^-^NKG2D^+^ and/or TCRαβ^+^CD3^+^CD4^-^CD8^-^NKG2D^-^ iDNT cells were selected using flow cytometry sorting.

### Evaluation of the Effects of iDNT Cells on Naïve B Cells *In Vitro*

To evaluate the effects of iDNT cells on B cells, freshly sorted NKG2D^+^ or NKG2D^-^iDNT cells were cocultured with different ratios of B cells for 18 hours with 5 ng/mL LPS in B cell medium (RPMI medium containing 10% FBS, 100 IU/ml penicillin, 100 μg/mL streptomycin, 2 mM L-glutamine, and 2 μg/mL anti-CD40 antibody). Apoptosis kits (Invitrogen, Catalog# V13241) were used to detect B cell apoptosis. The EdU Flow Cytometry Assay Kit (Invitrogen, Catalog# C10424) was used to analyze the proliferation of B cells in these mixed lymphocyte reactions. To observe the effects of NKG2D^+^ or NKG2D^-^ iDNT cells on naïve B cells and plasma cells, 10 ng/mL LPS was added to the B cell medium for 2 days. Anti-B220, anti-CD138, anti-CD86 and annexin V antibodies were used to analyze the mixed lymphocyte reaction.

### q-PCR

Total RNA was extracted from freshly sorted NKG2D^+^ iDNT cells or NKG2D^-^ iDNT cells using TRIzol reagent (Invitrogen, Catalog# 15596018) according to a standard protocol. The extracted RNA was reverse transcribed into cDNA, and q-PCR was performed to detect the expression of *Inf-γ*, *perforin*, *IL-17a*, *IL-2*, *IL-4*, *IL-10*, *IL-21* and *granzyme b.* The following primers were used for q-PCR: *Inf-γ* (forward: ATGAACGCTACACACTGCATC, reverse: CCATCCTTTTGCCAGTTCCTC), *perforin* (forward: AGCACAAGTTCGTGCCAGG, reverse: GCGTCTCTCATTAGGGAGTTTTT), *IL-17a* (forward: TTTAACTCCCTTGGCGCAAAA, reverse: CTTTCCCTCCGCATTGACAC), *IL-2* (forward: TGAGCAGGATGGAGAATTACAGG, reverse: GTCCAAGTTCATCTTCTAGGCAC), *IL-4* (forward: GGTCTCAACCCCCAGCTAGT, reverse: GCCGATGATCTCTCTCAAGTGAT), *IL-10* (forward: GCTCTTACTGACTGGCATGAG, reverse: CGCAGCTCTAGGAGCATGTG), *IL-21* (forward: GGACCCTTGTCTGTCTGGTAG, reverse: TGTGGAGCTGATAGAAGTTCAGG), and *granzyme b* (forward: CCACTCTCGACCCTACATGG, reverse: GGCCCCCAAAGTGACATTTATT). RNA extracted from B cells stimulated with LPS (10 μg/mL) in B cell medium for 12 hours was also reverse transcribed to detect *H60* (forward: CTGAGCTATCTGGGGACCATAC, reverse: AGTCTTTCCATTCACTGAGCAC), *Rae1* (forward: TTTGGGAGCACAACCACAGAT, reverse: TAAAGTTGGCGGGCTGAAAGA), *Mult1* (forward: CTGCCAGTAACAAGGTCCTTTC, reverse: GCTGTTCCTATGAGCACCAATG) and *GAPDH* (forward: AGGTCGGTGTGAACGGATTTG, reverse: TGTAGACCATGTAGTTGAGGTCA) using q-PCR following the standard protocol provided by the manufacturer.

### Statistical Analysis

All statistical analyses were performed using GraphPad v6.0 software. Data are presented as the means ± standard deviation. Student’s *t*-test was used to compare two independent variables (ns, not significant, *p < 0.05, **p < 0.01, and ***p < 0.001).

## Results

### MHC-II Impedes the Induction of CD4^+^CD127^hi^CD25^-^ T Cells Into iDNT Cells

**Figure 1:**
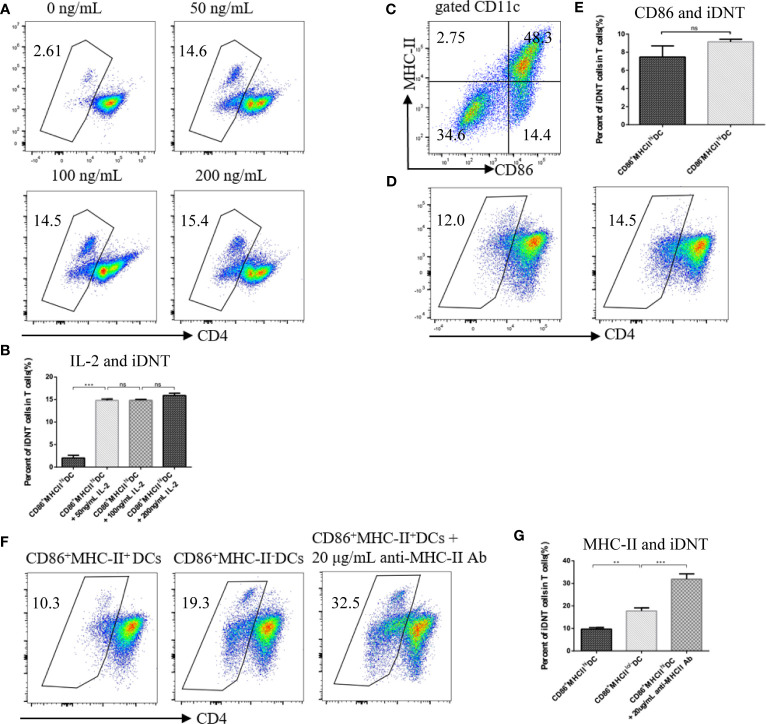
MHC class II impedes the induction of nonregulatory CD4^+^ T cells into iDNT cells. **(A, B)** Freshly sorted CD4^+^CD127^hi^CD25^-^ T cells were cocultured with mature bone marrow dendritic cells (CD86^-^MHC-II^+^ DCs) at a ratio of 1×10^5^ T cells to 2.5×10^4^ DCs for up to 7 days in 96-well round-bottom plates in complete RPMI 1640. Different concentrations of IL-2 (0, 50 ng/mL, 100 ng/mL or 200 ng/mL) were added to the culture system. TCRαβ^+^CD3^+^CD4^-^CD8^-^ iDNT cells were analyzed to evaluate the influence of IL-2 on induction. **(A)** The TCRαβ^+^CD3^+^CD4^-^CD8^-^ cell population is gated. Representative flow cytometry profiles of different concentrations of IL-2 (0, 50 ng/mL, 100 ng/mL or 200 ng/mL). **(B)** The bar graphs are representative of three independent experiments. **(C)** Representative data of the use of anti-CD86 and anti-MHC-II antibodies to divide bone marrow DCs into two subgroups: mature DCs (CD86^+^MHC-II^+^) and immature DCs (CD86^-^MHC-II^+^, CD86^+^MHC-II^-^ or CD86^-^MHC-II^-^). **(D, E)** Immature DCs (CD86^-^MHC-II^+^) or mature DCs (CD86^+^MHC-II^+^) were cocultured with freshly sorted nonregulatory CD4^+^ T cells. IL-2 (50 ng/mL) was added to the culture system. iDNT cells were analyzed to evaluate the influence of CD86 on induction. **(D)** Representative flow cytometry profiles of these two mixed lymphocyte reactions. **(E)** The bar graphs are representative of three independent experiments. **(F, G)** Immature DCs (CD86^+^MHC-II^-^), mature DCs (CD86^+^MHC-II^+^) or mature DCs with anti-MHC-II antibody (20 μg/mL) were cocultured with freshly sorted nonregulatory CD4^+^ T cells. IL-2 (50 ng/mL) was added to the culture system. iDNT cells were analyzed to evaluate the influence of MHC class II on induction. **(F)** Representative flow cytometry profiles of these three mixed lymphocyte reactions. **(G)** The bar graphs are representative of three independent experiments. Student’s *t*-test was used to compare two independent variables (ns, not significant, **p < 0.01, and ***p < 0.001).

To examine the factors that influence the induction of nonregulatory CD4^+^ T cells into TCRαβ^+^CD4^-^CD8^-^ DNT cells, we added different concentrations of IL-2 to a culture system (**Figures 1A, B**). We found that the addition of 50 ng/mL, 100 ng/mL or 200 ng/mL IL-2 greatly affected the number of iDNT cells compared to no IL-2 treatment (**Figure 1B**). To further examine whether immature bone marrow DCs also induced this process, CD86^-^MHC-II^+^ DCs were cocultured with nonregulatory CD4^+^ T cells (**Figure 1C**). We found no significant difference between CD86^+^MHC-II^+^ DCs and CD86^-^MHC-II^+^ DCs (**Figure 1D**). CD86^+^MHC-II^lo/-^ DCs were also used. We found that low or no MHC expression on CD86^+^ DCs influenced the induction process (**Figures 1E, F**), and the addition of an anti-MHC-II antibody to the CD86^+^MHC-II^+^ DC group significantly increased the number of iDNT cells in the group compared to the numbers in other groups (**Figures 1E–G**).

### IL-2 and MHC-II May Regulate NKG2D Expression on iDNT Cells

**Figure 2:**
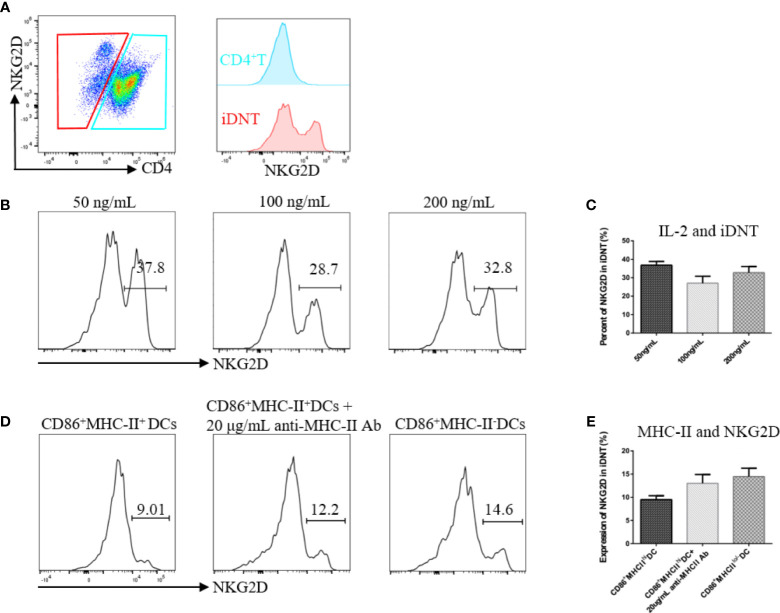
IL-2 and MHC-II may regulate NKG2D expression on iDNT cells. **(A)** A representative experiment shows that a group of iDNT cells expressed NKG2D molecules. **(B, C)** Nonregulatory CD4^+^ T cells were cocultured with mature bone marrow dendritic cells (CD86^-^MHC-II^+^ DCs). IL-2 (50 ng/mL, 100 ng/mL or 200 ng/mL) was added to the mixed lymphocyte reaction. After successful induction, the percentage of NKG2D on iDNT cells was detected using flow cytometry. **(B)** Histograms are representative of NKG2D expression on iDNT cells. **(C)** The bar graphs are representative of three independent experiments. **(D, E)** Immature DCs (CD86^+^MHC-II^-^), mature DCs (CD86^+^MHC-II^+^) or mature DCs with anti-MHC-II antibody (20 μg/mL) were cocultured with nonregulatory CD4^+^ T cells. IL-2 (20 ng/mL) was added to the mixed lymphocyte reaction. After successful induction, the percentage of NKG2D on iDNT cells was analyzed using flow cytometry. **(D)** Histograms are representative of NKG2D expression on iDNT cells. **(E)** The bar graphs are representative of three independent experiments.

After the successful induction of iDNT cells, we found that some of these cells expressed NKG2D molecules (**Figure 2A**). Different concentrations of IL-2 and immature DCs revealed that IL-2 and MHC-II were related to NKG2D expression on iDNT cells (**Figures 2B, E**). CD86^+^ DCs with low or no MHC expression appeared to induce more iDNT cells than other types of DCs (**Figures 2B, E**).

### NKG2D Enhances iDNT Cell-Mediated Inhibition of B Cell Proliferation and Promotes B Cell Apoptosis

**Figure 3:**
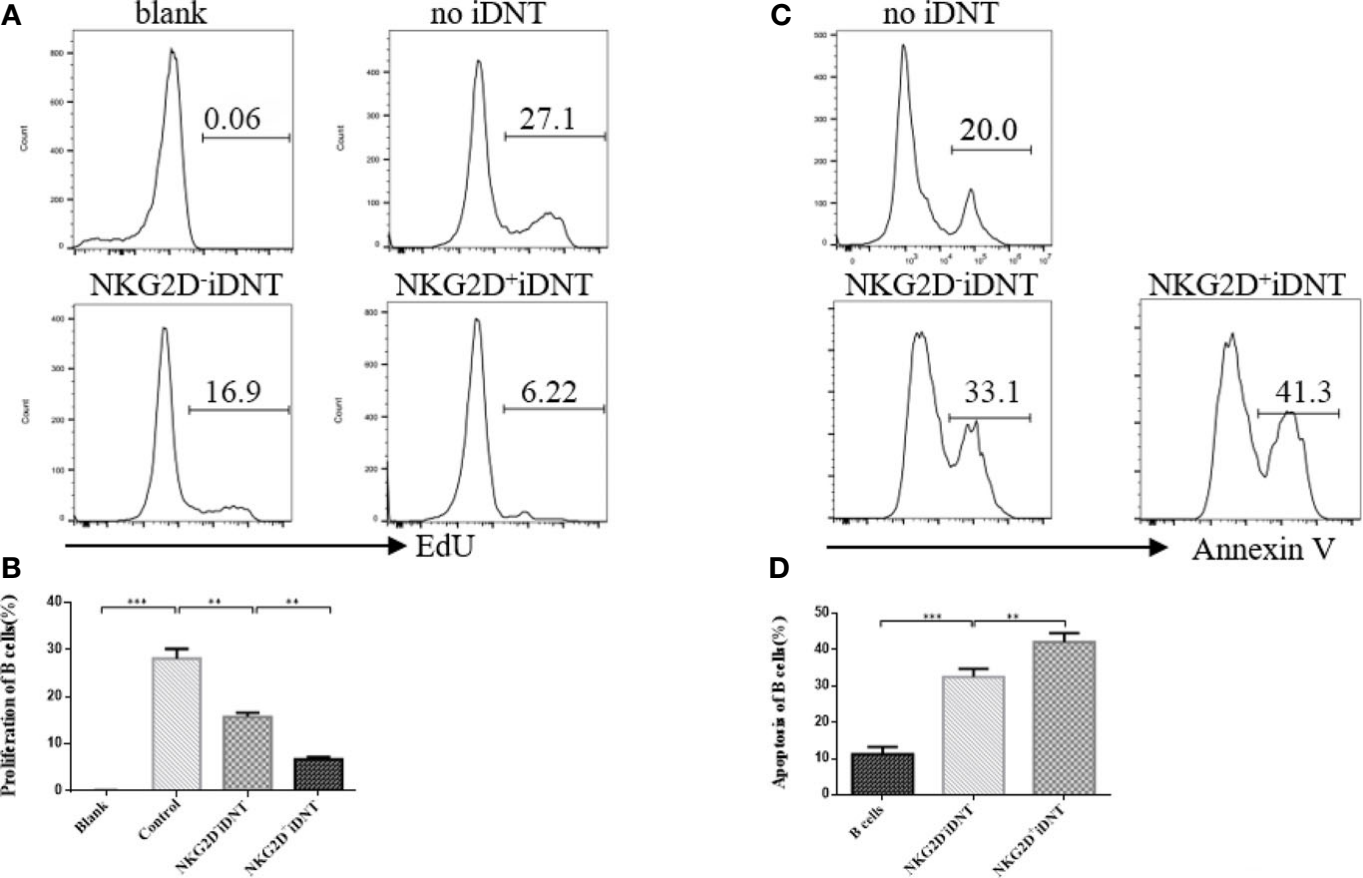
NKG2D enhances iDNT cell-mediated inhibition of B cell proliferation and promotes B cell apoptosis. Naïve B cells were cultured with sorted TCRαβ^+^CD3^+^CD4^-^CD8^-^NKG2D^+^ or TCRαβ^+^CD3^+^CD4^-^CD8^-^NKG2D^-^ iDNT cells at a ratio of 4×10^5^ B cells to 1×10^5^ iDNT cells in 96-well round-bottom plates in B cell medium. EdU (10 μM) and LPS (5 μg/mL) were added to the medium, and B cell proliferation and apoptosis were detected approximately 18 hours later. **(A)** Histograms are representative of B cell proliferation. **(B)** The bar graphs are representative of three independent B-cell proliferation experiments. **(C)** Histograms are representative of B cell apoptosis. **(D)** The bar graphs are representative of three independent B-cell apoptosis experiments. Student’s *t*-test was used to compare two independent variables (**p < 0.01 and ***p < 0.001).

To study the effects of NKG2D expression on iDNT cells on B cell proliferation and apoptosis, we sorted NKG2D^+^ and NKG2D^-^ iDNT cells and incubated these cells with naïve B cells at a ratio of 4×10^5^ B cells to 1×10^5^ iDNT cells in 96-well round-bottom plates in B cell medium (**Figures 3A, B**). EdU (10 μM) and LPS (5 μg/mL) were added to the medium, and B cell proliferation and apoptosis were detected approximately 18 hours later. We found that NKG2D^+^ iDNT cells had a stronger ability to inhibit B cell proliferation and promote B cell apoptosis than NKG2D^-^ iDNT cells. NKG2D^-^ iDNT cells also suppressed B cell proliferation and promoted B cell apoptosis (**Figures 3C, D**).

### NKG2D Enhances iDNT Cell-Mediated Inhibition of B Cell Differentiation Into Plasma Cells *In Vitro*

**Figure 4:**
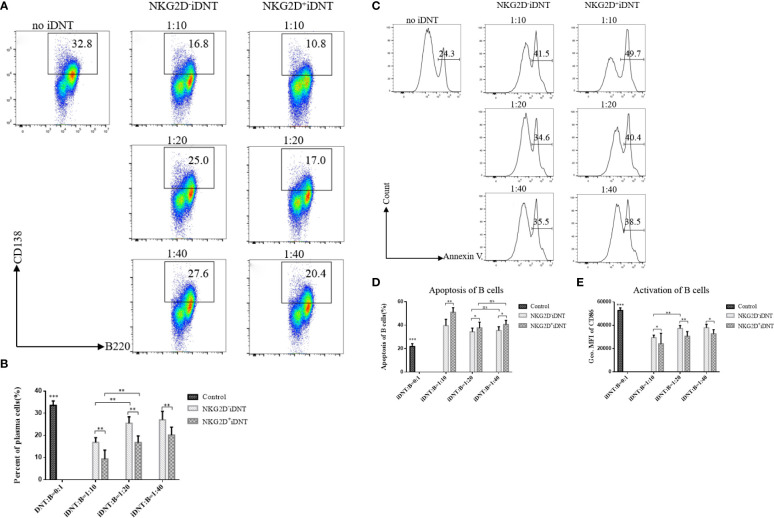
NKG2D enhances iDNT cell-mediated inhibition of B cell differentiation into plasma cells *in vitro*. Freshly sorted TCRαβ^+^CD3^+^CD4^-^CD8^-^NKG2D^+^ or TCRαβ^+^CD3^+^CD4^-^CD8^-^NKG2D^-^ iDNT cells were incubated with naïve B cells at a gradient ratio of 0.4×10^5^, 0.2×10^5^ or 0.1×10^5^ iDNT cells to 4×10^5^ B cells in 96-well round-bottom plates in B cell medium for 2 days. LPS (10 μg/mL) was added to the medium, and we analyzed the activation, differentiation and apoptosis of B cells. **(A)** Representative flow cytometry profiles of B cell differentiation into plasma cells *in vitro*. **(B)** The bar graphs are representative of three independent B-cell differentiation experiments. **(C)** Histograms are representative of B cell apoptosis at different ratios. **(D)** The bar graphs are representative of three independent B-cell apoptosis experiments. **(E)** The bar graphs are representative of three independent B-cell activation experiments. Student’s *t*-test was used to compare two independent variables (ns, not significant, *p < 0.05, **p < 0.01, and ***p < 0.001).

To further evaluate the function of NKG2D molecules, we extended the incubation time to 2 days, added a higher concentration of LPS (10 μg/mL) to the medium and reduced the number of B cells to a gradient ratio of 4×10^5^ B cells to 0.4×10^5^, 0.2×10^5^ or 0.1×10^5^ iDNT cells. We analyzed B cell differentiation and found that NKG2D^+^ iDNT cells had a stronger inhibitory ability than NKG2D^-^ iDNT cells at all ratios (**Figures 4A, B**). We also detected B cell apoptosis and found that NKG2D^+^ iDNT cells promoted more B cell apoptosis than NKG2D^-^ iDNT cells at all ratios. However, the difference in apoptosis between groups became less obvious as the ratio decreased (**Figures 4C, D**). We further evaluated CD86 to detect the level of activated B cells. As expected, iDNT cells suppressed B cell activation, and NKG2D^+^ iDNT cells had a stronger inhibitory capability than NKG2D^-^ iDNT cells (**Figure 4E**).

### NKG2D^+^ iDNT Cells Express Higher Levels of Granzyme B Than NKG2D^-^ iDNT Cells, and Activated B Cells Express NKG2D Ligands

**Figure 5:**
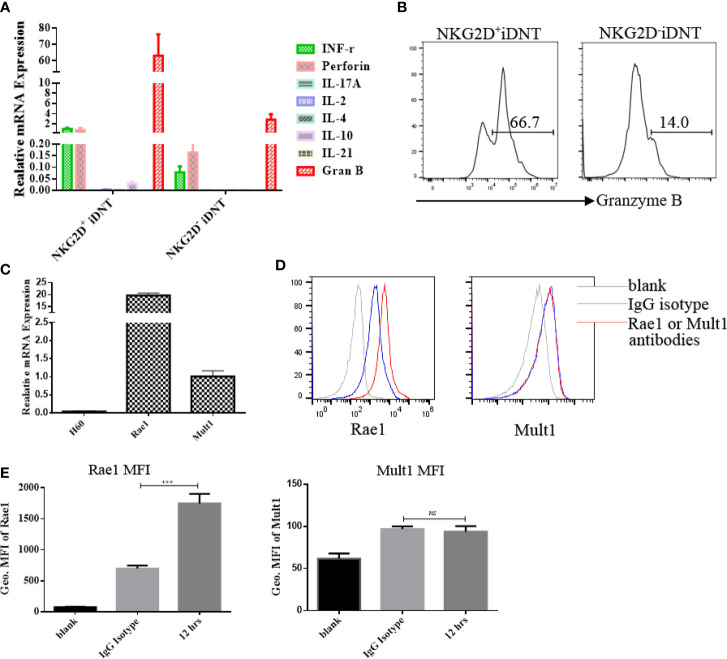
NKG2D^+^ iDNT cells express higher levels of granzyme B than NKG2D^-^ iDNT cells, and activated B cells express the NKG2D ligand Rae1. **(A)** Q-PCR was performed to examine granzyme B mRNA levels in freshly sorted TCRαβ^+^CD3^+^CD4^-^CD8^-^NKG2D^+^ and TCRαβ^+^CD3^+^CD4^-^CD8^-^NKG2D^-^ iDNT cells. **(B)** Granzyme B protein was detected using flow cytometry. **(C)** Naïve B cells were stimulated with LPS in B cell medium for 12 hours, and q-PCR was performed to examine the expression levels of NKG2D ligands. **(D)** Naïve B cells were stimulated with LPS in B cell medium for 12 hours, and flow cytometry was performed to examine the expression of Rae1 and Mult1. **(E)** Geometric MFI of Rae1 and Mult1 protein. Student’s t-test was used to compare two independent variables (ns, not significant, ***p < 0.001).

To clarify the mechanisms of NKG2D^+^ DNT cell inhibition of B cell function, q-PCR was performed to examine granzyme B mRNA levels. We found that NKG2D^+^ iDNT cells and NKG2D^-^ iDNT cells expressed granzyme B, but NKG2D^+^ iDNT cells expressed higher levels of granzyme B than NKG2D^-^ iDNT cells (**Figures 5A, B**). This result is consistent with previous studies that showed that iDNT cells also expressed perforin. To determine whether activated B cells expressed NKG2D ligands, we used q-PCR to detect H60, Rae1 and Mult1. We found that activated B cells exhibited Rae1 expression (**Figures 5C, D**).

## Discussion

During antigen-presenting cell (APC) education, CD4^+^ T cells are activated, proliferate, differentiate and secrete cytokines. All of these immune functions depend on at least two interactive signals between two types of cells: the first signal is derived from TCR-CD3 complexes, which are expressed on the surface of CD4^+^ T cells and combine with peptide-MHC-II on APCs when activated; the second signal is transduced *via* CD86/80 molecules, which interact with CD28 molecules. Although several types of cells express MHC-II molecules, DCs are one of the most important APCs expressing this molecule and participating in CD4+ T cell immune responses. Once CD4^+^ T cells recognize a foreign peptide-MHC-II complex on the plasma membrane of DCs, these cells form TCR-CD3-peptide-MHC-II complexes. CD4 molecules expressed on CD4^+^ T cells restructure themselves and extend synapses with MHC-II molecules. Our study found that MHC-II^lo/-^CD86^+^ DCs, but not MHC-II^+^CD86^+^ DCs, enhanced induction. The addition of an anti-MHC-II neutralizing antibody to the CD86^+^MHC-II^+^ DC group significantly increased the number of iDNT cells (**Figures 1E, F**). These results suggested that CD4 molecules expressed on CD4^+^ T cells “disappeared” because of low or nonexistent MHC-II expression. Unlike CD4^+^ T cells binding to liver cell MHC-II molecules to form TCR-CD3-peptide-MHC-II complexes, which are later removed from liver cells in autoimmune hepatitis (24), MHC-II^lo/-^CD86^+^ DCs cannot efficiently contact CD4^+^ T cells. Therefore, CD4 molecules cannot extend synapses with MHC-II molecules and may be gradually lost during the process of cell proliferation supported by a second signal and IL-2. Unlike many receptors that are internalized after continuous stimulation, the CD4 molecule present on the surface of CD4^+^ T cells do not disappear *via* internalization (11). A recent study reported that concomitant disruption of the CD4 and CD8 genes facilitated the development of DNT cells in the periphery (25). This study used transgenic mice expressing human HLA class II molecules, HLA-DR3 or HLA-DQ8, as test subjects. Because human HLA class II molecules present superantigens more efficiently than murine MHC class II molecules, murine MHC class I and II molecules did not efficiently contact the coreceptors CD4/CD8, which led to CD4 and CD8 gene inactivation. These authors found that this disruption enabled the development of DNT cells in the periphery, which is consistent with our findings.

Numerous studies reported that IL-2 was an important factor that promoted robust proliferation of CD4^+^ T cells after interactions with DCs (26–28). The proliferative ability of treated cells increased gradually with increasing IL-2 concentrations. We also added IL-2 during the process of induction based on our previous studies (11, 12), but we found that different amounts of IL-2 did not obviously change induction (**Figures 1A, B**). Although DCs secrete IL-2 (27, 29, 30), this secretion is not sufficient. These results indicate that induction requires exogenous IL-2. Critically, anti-CD3 antibodies could not be added to the culture system when inducing CD4 molecule downregulation. This observation is very important because no iDNT cells were observed when anti-CD3 antibodies were added to the mixed lymphocyte reaction (unpublished data).

NKG2D is an activating receptor that is commonly expressed on NK cells. NKG2D is also present on NKT cells, activated CD8^+^ T cells, γδ T cells, macrophages, and a small subgroup of CD4^+^ T cells (20, 31, 32). However, little is known about whether TCRαβ^+^CD3^+^CD4^-^CD8- T cells express NKG2D. Our study found that a group of iDNT cells expressed NKG2D molecules (**Figure 2A**), and the addition of IL-2 increased induction and NKG2D expression (**Figures 2D, E**). These results suggested that our iDNT cells were activated after successful induced. This result is similar to the results achieved with IL-2 administration, which activated CD8^+^ T cells and increased the expression of NKG2D. We do not understand why MHC-II influenced the expression of NKG2D on iDNT cells (**Figures 2B, C**). However, we will study the mechanism of MHC-II influence on NKG2D in future work.

Activation of the NKG2D receptor promoted NK cell killing activity. This receptor enhanced effector and memory CD8^+^ T cell formation (21, 22). Our study found a group of iDNT cells, NKG2D^+^ iDNT cells, that promoted more B cell apoptosis and a stronger inhibition of B cell proliferation and plasma cell formation than NKG2D^-^ iDNT cells (**Figures 3**, **4**). These results demonstrated that NKG2D enhanced iDNT cell-mediated regulation of B cells. We also determined why NKG2D^+^ iDNT cells had a stronger regulatory function than NKG2D^-^ iDNT cells. In this study, we found naïve B cells upregulated the protein expression of the NKG2D ligand upon LPS stimulation (**Figures 5D, E** and **Supplementary Figures 1A–C**), which is consistent with a previous study that B cells were significantly stained with the NKG2D tetramer after stimulation of splenocytes with ConA or LPS (33). Our study also found that the transcript of Mult1 but not posttranslational protein was detected (**Figures 5C–E**), indicating the existence of translational or posttranslational regulation. A previous study reported that Mult1 protein was ubiquitinated and degraded under normal conditions (34). However, the degradation and ubiquitination was reduced in response to cell stress (34). In our research, stimulation of naïve B cells with LPS for 12 hours might not be sufficient to reduce the degradation and ubiquitination of Mult1 (**Supplementary Figures 1D, E**). NKG2D^+^ iDNT cells and NKG2D^-^ iDNT cells expressed granzyme B, and NKG2D^+^ iDNT cells produced more granzyme B than NKG2D^+^ iDNT cells (**Figures 5A, B**). These results suggest that NKG2D^-^ iDNT cells are previously activated and secrete some granzyme B. NKG2D activation enhances NKG2D^+^ iDNT cell regulation *via* NKG2D ligands on B cells by inducing an increased release of granzyme B. A recent study showed that the levels of NKG2D ligand expression on splenic B cells increased in mice with aging (35). Another study reported that a small subgroup of mouse B cells, B1a cells, which exhibit NKG2D and NKG2D deficiency, impaired B1a cell development and T cell-independent immune responses (36). All of these reports demonstrate that NKG2D regulates B cell development and effector B cells.

**Figure 6:**
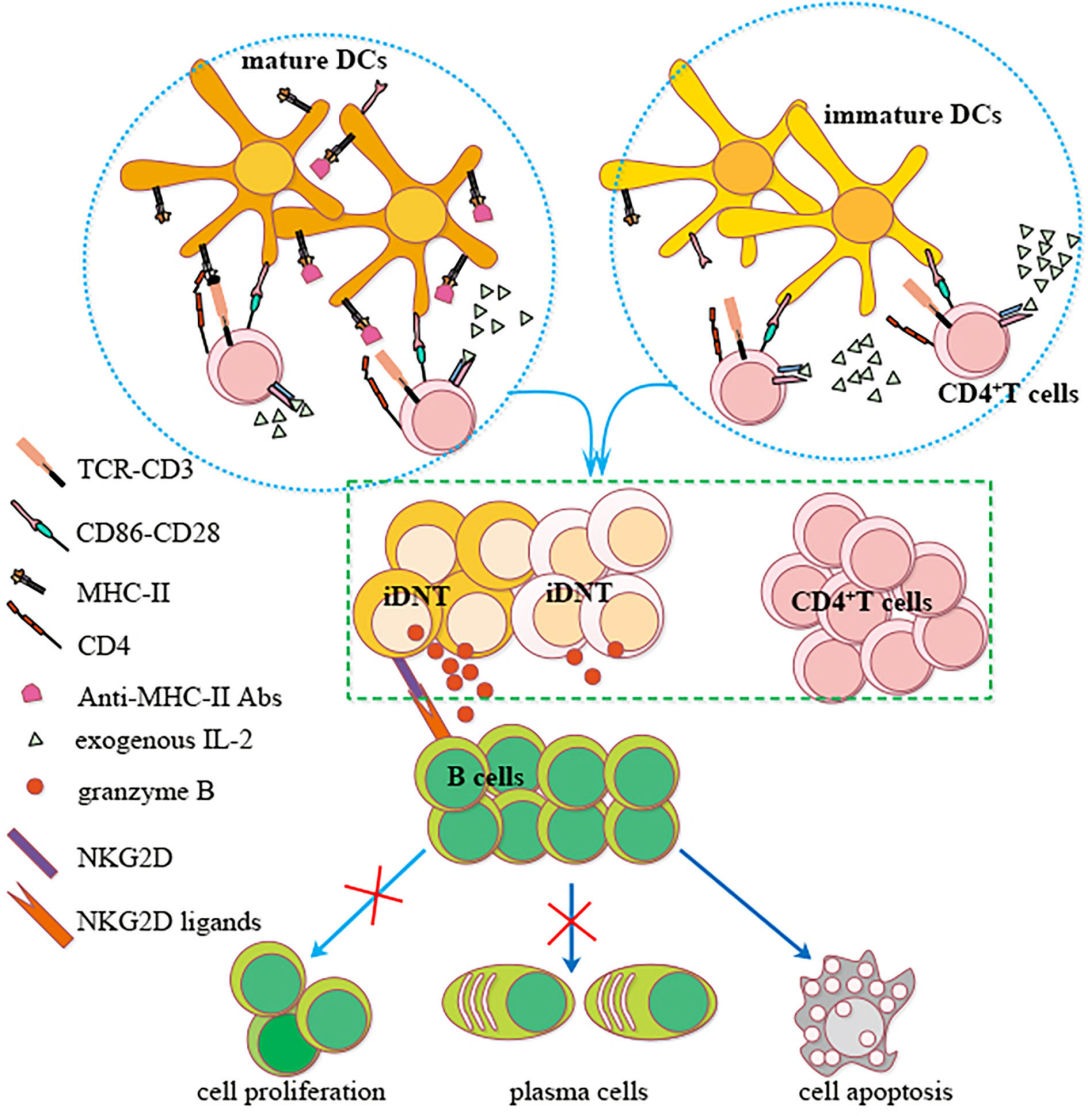
A possible mechanism of nonregulatory CD4^+^ T cells induction into iDNT cells and NKG2D enhancement of iDNT cells regulation of B cells. Nonregulatory CD4^+^ T cells may be induced to become regulatory iDNT cells *in vitro*. IL-2 promotes the induction process, and MHC-II expressed on bone marrow DCs impedes this process. These iDNT cells were activated after successful induction, which promoted B cell apoptosis and inhibited B cell proliferation and plasma cell formation. A small portion of iDNT cells express NKG2D, which induces the release of granzyme B to enhance iDNT cell-mediated regulation of B cell functions *via* the NKG2D ligand Rae1.

In summary, we showed that nonregulatory CD4^+^ T cells may be induced to become regulatory iDNT cells *in vitro*. IL-2 promoted the induction process, and MHC-II expression on bone marrow DCs impeded this process. These iDNT cells were activated after successful induction, which promoted B cell apoptosis and inhibited B cell proliferation and plasma cell formation. A small portion of iDNT cells expressed NKG2D, which induced the release of granzyme B to enhance iDNT cell-mediated regulation of B cell functions *via* NKG2D ligands (**Figure 6**). Therefore, our research provides insight for understanding the mechanism of peripheral immune tolerance and the development of a potential treatment for chronic allograft rejection.

## Data Availability Statement

The raw data supporting the conclusions of this article will be made available by the authors, without undue reservation.

## Ethics Statement

The animal study was reviewed and approved by Peking University People’s Hospital Animal Ethics and Experimental Committee.

## Author Contributions

Conceived and designed the experiments: S-hH and J-yZ. Performed the experiments: S-hH. Analyzed the data: L-hZ. Contributed reagents/materials/analysis tools: J-cG, JG, X-dX, QC, and XX. Wrote the article: S-hH and ZL. All authors contributed to the article and approved the submitted version.

## Funding

This study was supported by grants from the National Natural Science Foundation of China (No. 81570590 and 81502509).

## Conflict of Interest

The authors declare that the research was conducted in the absence of any commercial or financial relationships that could be construed as a potential conflict of interest.

## Supplementary Material

Supplementary Figure 1 The mRNA and Protein expression of Rae1 and Mult1. **(A–E)** Naïve B cells were stimulated with LPS (10 μg/mL) in B cell medium for 0, 6, 18 and 48 hours. Flow cytometry was performed to examine the expression of Rae1 and Mult1. **(A)** The protein expression of Rae1 was upregulated with the extension of culture time. **(B)** Geometric MFI of Rae1. **(C)** Percent of Rae1 protein expression with culture time. **(D)** The protein expression of Mult1. **(E)** Geometric MFI of Mult1. **Figures 1A–E** is an independent experiment. Student’s t-test was used to compare two independent variables (ns, not significant, *p < 0.05, **p < 0.01, and ***p < 0.001).
