# Supplementary figures and images for: NKG2D Enhances Double-Negative T Cell Regulation of B Cells
Source: Front Immunol. 2021 Jun 16;12:650788. doi: 10.3389/fimmu.2021.650788 (PMC8242353; doi:10.3389/fimmu.2021.650788)

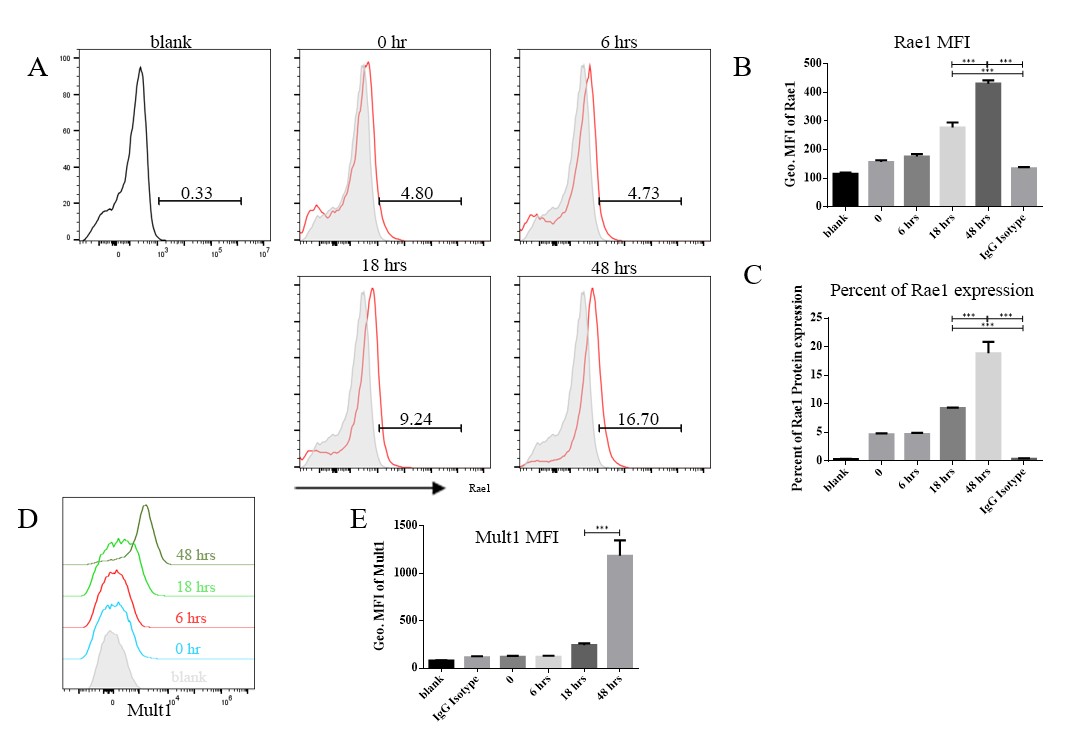

Supplement: Supplementary Figure 1 — The mRNA and Protein expression of Rae1 and Mult1. (A–E) Naïve B cells were stimulated with LPS (10 μg/mL) in B cell medium for 0, 6, 18 and 48 hours. Flow cytometry was performed to examine the expression of Rae1 and Mult1. (A) The protein expression of Rae1 was upregulated with the extension of culture time. (B) Geometric MFI of Rae1. (C) Percent of Rae1 protein expression with culture time. (D) The protein expression of Mult1. (E) Geometric MFI of Mult1. Figures 1A–E is an independent experiment. Student’s t-test was used to compare two independent variables (ns, not significant, *p < 0.05, **p < 0.01, and ***p < 0.001). [file Image_1.tif]
